# Subsidence in Centraliser Sign: A Novel Tool to Help Predict Early Subsidence in Periprosthetic Femoral Fractures Around Polished Tapered Stems Treated With Internal Fixation

**DOI:** 10.7759/cureus.42742

**Published:** 2023-07-31

**Authors:** Suroosh Madanipour, Prashant Singh, Arpit Patel, Ruqqayyah Beg, Menazir Sha, Ishvinder S Grewal, Farhad Iranpour, Padmanabhan Subramanian

**Affiliations:** 1 Trauma and Orthopaedics, Royal Free London NHS Trust, London, GBR; 2 Trauma and Orthopaedics, University College London Medical School, London, GBR; 3 Orthopaedic Surgery, University of Texas (UT) Southwestern Medical Center, Dallas, USA

**Keywords:** open reduction internal fixation, revision hip arthroplasty, cemented total hip arthroplasty, periprosthetic hip fracture, periprosthetic fracture

## Abstract

Background

When treating periprosthetic femoral fractures (PPF) around polished taper slip stems (PTS), determining which patients can be successfully treated with internal fixation can be challenging. We have described the subsidence-in-centraliser (SINC) sign as a radiographic feature of PPF around PTS stems. We hypothesise that a positive SINC sign can help predict a poorer outcome for the fixation of these fractures.

Patients and methods

Retrospective identification of PPFs around cemented PTS with an appreciable centraliser on radiographs was conducted at a single centre. A positive SINC sign was defined as a post-injury radiograph demonstrating >50% reduction in the radiographic lucency representing the stem centraliser when compared to pre-injury films or complete obliteration of distal lucency when no pre-injury film was available. The primary outcome was the rate of subsequent stem subsidence on follow-up radiographs comparing SINC-positive and SINC-negative fractures, which were managed with open reduction and internal fixation (ORIF).

Results

Fifty-four patients were included in the analysis. The mean age was 76.8 years, and the mean follow-up for all patients was 12.7 months. Thirty-five fractures were deemed SINC-positive, and 19 were SINC-negative. 17/17 (100%) SINC-positive fractures managed with fixation underwent further subsidence (mean 5.4 mm, SD 2.8). A positive SINC sign demonstrated a sensitivity of 90.5% and specificity of 100% for subsequent stem subsidence in fractures treated without revision. SINC positive fractures underwent significantly more subsidence compared with SINC negative fractures when fixed (5.4 mm vs. 0.28 mm, U = 6.50, p<0.001) at a mean follow-up of 12.7 months. The SINC sign demonstrated strong inter- (k=0.96) and intra-rater (k=0.86) reliability.

Conclusion

The SINC sign can serve as a useful adjunct in the decision to fix or revise PPF around PTS. A positive SINC sign may represent a cement mantle that cannot be reconstituted anatomically, leading to subsidence after treatment with ORIF.

## Introduction

Periprosthetic hip fractures (PHF) are common post-operative complications following total hip arthroplasty (THA) [1,2]. PHFs are projected to rise in prevalence alongside an ageing population with increased demands and an increasing prevalence of THA [3,4]. PHFs are associated with a high rate of reoperation and mortality [5-7].

The Vancouver classification [8] and subsequent unified classification systems [9] are used internationally to guide treatment strategies. These demonstrate good inter- and intra-observer reliability and validity [10]. The classification, however, does not always help in differentiating between B1 and B2 fractures, i.e., whether a stem is loose. This distinction is a key determinant of management: B1 fractures can be successfully treated with open reduction and internal fixation (ORIF) [11], whereas B2 fractures around loose stems can be appropriately treated with revision arthroplasty [12]. However, failure to make this distinction can lead to poor outcomes when B2 fractures are treated with ORIF instead of revision [13]. This ‘misclassification’ of B1 fractures can contribute to poorer outcomes for B1 fractures compared with B2 and B3 [14].

High failure rates amongst B1 fractures treated with ORIF have been attributed to the underdiagnosis of loose implants [15-17]. Lindahl et al. determined that 47% of PHF stems were ‘unknown loose’ based on pre-operative radiographs [16].

Critically, the Vancouver classification does not distinguish between types of stem fixation. Fractures around cemented stems account for a large volume of PHFs globally [18]. Polished tapered stems (PTS) enable controlled subsidence into the femoral canal and cement the mantle as a wedge, allowing for radial hoop stresses to transfer force to the bone. Fractures around PTS, compared with cementless or composite beam-type stems, behave differently in that there is no "fixation" between the implant and the cement interface [19].

Modern PTS has a distal hollow centraliser allowing for a central position of the stem [20] and consequently a more equal force distribution within the cement mantle and controlled subsidence. This hollow centraliser design, initially introduced in the 1980s to prevent distal cement ‘punch-out’ fractures [21], has become central to stem design [22].

We have previously described a radiographic sign in fractures around cemented polished tapered stems and termed this the ‘SINC’ sign, referring to ‘subsidence-in-centraliser’, whereby a fracture can cause the stem to acutely subside into the centraliser [23].

We hypothesise that a positive SINC sign is indicative of a cement mantle that cannot be adequately reconstituted and can therefore help to predict those fractures that will perform worse with internal fixation. Therefore, the central question of our study is whether the SINC sign is an indicator of poor performance with osteosynthesis in periprosthetic fractures around the THA.

## Materials and methods

Study design

**Figure 1:**
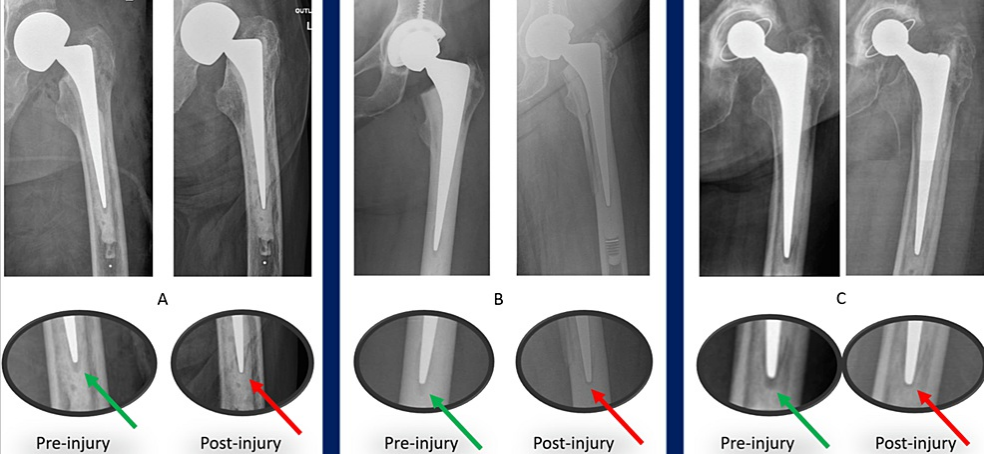
Complete obliteration AP radiographs of three separate patients (A-C) demonstrating pre-injury normal features after index arthroplasty and post-injury positive ‘SINC’ sign with complete obliteration of the lucency representing the centraliser.

**Figure 2:**
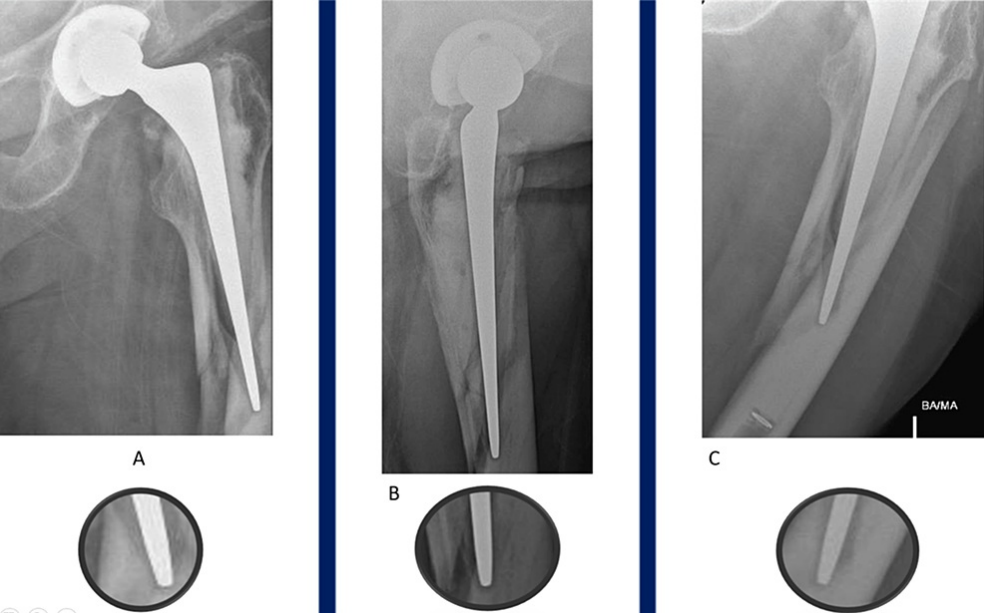
No pre-injury film available AP (A), lateral (B) and oblique (C) radiographs of one patient taken post-injury demonstrating positive ‘SINC’ sign with complete obliteration of the lucency representing the centraliser. No pre-injury film was available.

**Figure 3:**
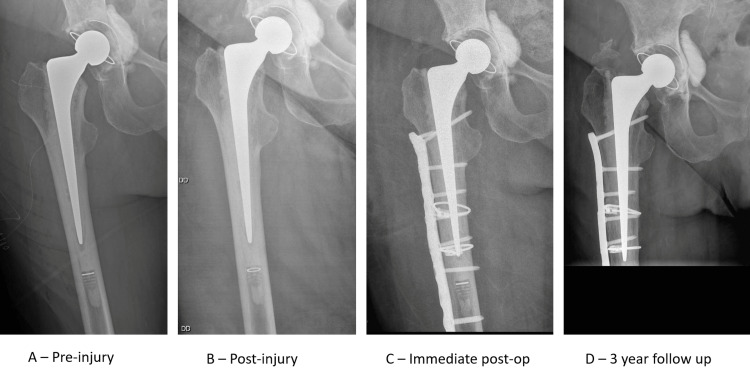
50% rule and further subsidence (A) Pre-injury AP radiograph; (B) AP radiograph taken post-injury demonstrating positive ‘SINC’ sign with >50% obliteration of lucency representing the centraliser when compared with pre-injury radiograph; (C) immediate post-operative radiograph demonstrating stem position following plate osteosynthesis; (D) AP radiograph at three year follow up demonstrating significant further stem subsidence.

A positive SINC sign was defined as a >50% reduction in the distal lucency representing the stem centraliser when compared to pre-fracture radiographs (Figures 1-3) or complete obliteration of this lucency when no pre-injury radiograph was available (Figure 2). This was measured as the linear distance from the tip of the stem to the end of the distal lucency.

Retrospective identification of all cases of periprosthetic hip fractures presenting to a single hospital system in the United Kingdom between January 2011 and December 2020 was performed. Available imaging and notes were reviewed using the electronic medical record (EMR) and picture archiving and communication system (PACS).

Patient selection

The inclusion criteria were patients with periprosthetic femoral fracture (PPF) around cemented stems whose radiographs demonstrated an appreciable centraliser on initial fracture and post-injury radiographs. Cases were excluded if the femoral stems had no appreciable centraliser on radiographs (e.g., Stanmore, Stryker, Kalamazoo, MI), if the stems displayed radiographic evidence of pre-injury loosening or osteolysis, if the stem was uncemented, or if a minimum follow-up radiograph of eight weeks was not available. Treatment was determined as either conservative (non-operative), ORIF, or revision arthroplasty and was decided upon following discussion amongst surgeons at a trauma meeting. ORIF was conducted by general orthopaedic trauma surgeons and revision by fellowship-trained hip arthroplasty surgeons.

Additionally, 100 patients with 107 consecutive cases of primary THA using PTS were identified from the trust coding database between January 2018 and April 2018, with no history of trauma and with an appreciable centraliser on radiographs. The presence or absence of the SINC sign was documented. The purpose of this was to determine whether a positive SINC sign is a normal finding in follow-up radiographs of PTS in the absence of trauma.

Data collection and analysis

Inter-observer reliability of the SINC sign was assessed by an independent review of all pre-operative radiographs by an arthroplasty and trauma consultant (PS) and a joint reconstruction fellow (FI). Cohen’s Kappa was used to determine the level of agreement between the two assessors’ ratings.

Intra-observer reliability was assessed by the review of radiographs by a senior resident (SM) on two separate occasions, separated by three weeks. Cohen’s Kappa was used to determine the level of agreement between ratings on each occasion [24]. For both measures, assessors were blinded to patient treatment and outcome to reduce observer bias.

Subsequent stem subsidence on follow-up radiographs was measured using digital templating software (TraumaCad, BrainLab, Inc., Westchester, IL) by two authors (PS and FI). The femoral head diameter was used for calibration of the magnification of the radiographs in the template. The vertical distance from the shoulder of the stem to the tip of the greater trochanter along the longitudinal femoral shaft axis was used to calculate the subsidence. Subsequent stem subsidence was measured at the final available follow-up radiograph in comparison to immediate post-injury radiographs. The degree of subsequent stem subsidence was compared between the SINC-positive and SINC-negative groups using the Mann-Whitney U test. The inter-observer reliability of the digital templating software method of measuring subsequent stem subsidence was calculated using the intraclass correlation coefficient (ICC).

To assess the accuracy of the SINC sign in predicting subsequent subsidence, sensitivity, and specificity were calculated using 2 × 2 contingency tables, with exact Clopper-Pearson confidence intervals calculated for 95%. A SINC-positive radiograph was defined as a positive test, and subsequent stem subsidence as a dichotomous outcome was defined as a true positive finding. This analysis was only applied to PHFs that were managed conservatively or with fixation as opposed to revision surgery. In addition, the Phi coefficient was calculated to assess the association between a SINC-positive radiograph and the rate of subsequent stem subsidence. Statistical analyses were conducted using IBM SPSS Statistics software, version 25 (IBM SPSS, Armonk, NY, USA). A p-value of <0.05 was considered statistically significant.

## Results

Of the 100 consecutive patients with no history of trauma who underwent 107 THA, two post-operative radiographs demonstrated a positive SINC sign with complete obliteration. This was evident at the initial post-operative radiographs and the latest follow-up. The remaining 105 THA had negative SINC signs at the initial post-operative and latest follow-up. The mean follow-up in this group was 19.6 months (range: 12-68 months).

**Figure 4:**
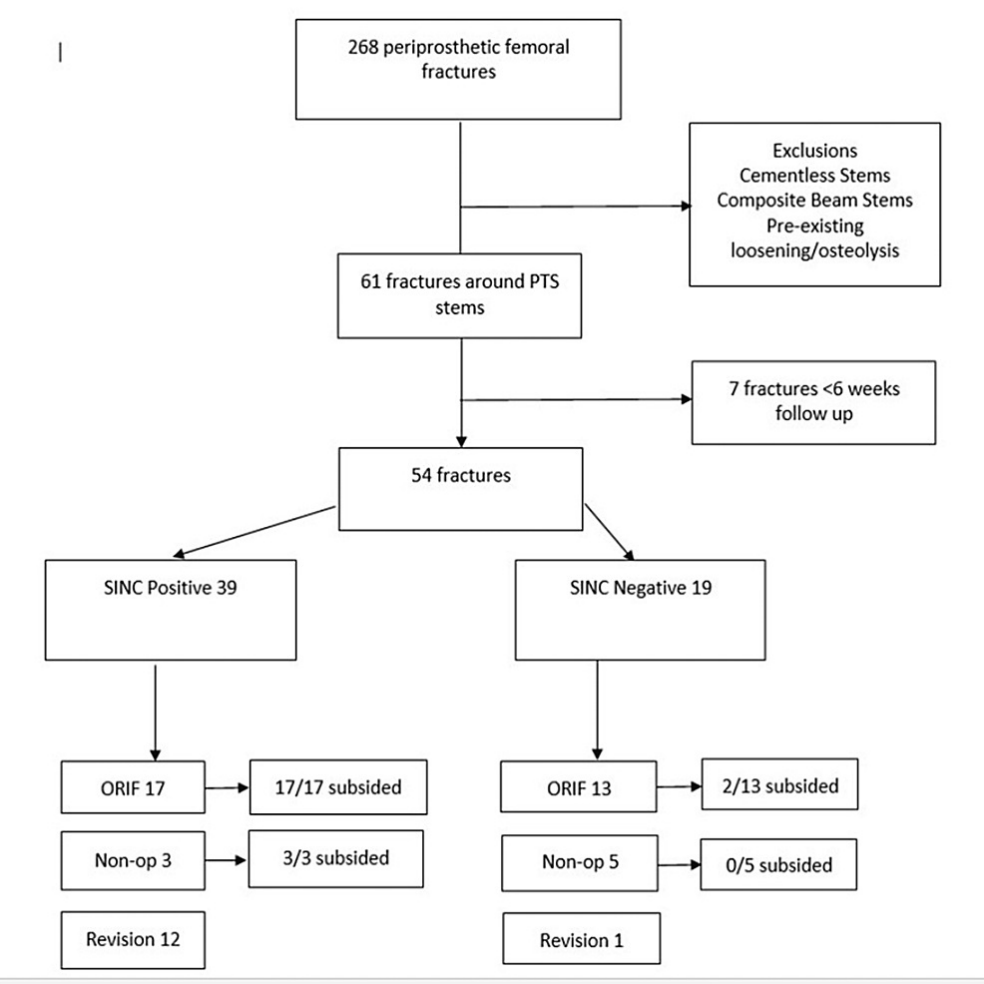
Flowchart

Out of 268 PHFs originally identified, following exclusions, a total of 54 patients with 54 PHFs were included in the analysis, all of which occurred around a cemented, polished, tapered stem with an appreciable centraliser on radiographs (Figure 4).

The mean age at injury was 76.8 years (SD 12.2). Thirty-two patients (67%) were female, and 17 (33%) were male. Forty-seven femoral stems were CPT (Zimmer, Warsaw IN, US), three were C-Stem (Depuy, Warsaw IN, US) and four were Exeter (Stryker, Kalamazoo, MI).

The mean follow-up of cases managed non-operatively or with fixation was 12.8 months (range 2-53, SD 10.3). The minimum follow-up was two months. The median time to surgery was three days for ORIF and six days for revision arthroplasty. Pre-injury radiographs were available for 25/37 patients managed conservatively or with fixation (67.6%).

SINC positive

Thirty-five PHFs were deemed to be ‘SINC’ positive (Table 1). Of these, two were managed conservatively due to significant patient co-morbidities and one because the surgical team felt the patient could be managed with a trial of conservative treatment. Fifteen of thirty-five (42.9%) were treated with revision arthroplasty to an uncemented distal fix taper fluted stem. Seventeen of thirty-five (48.9%) SINC-positive patients were treated with an open reduction and internal fixation (ORIF), which comprised 15 patients with a lateral locking plate, one with double plating, one with an additional strut graft, and two patients with cerclage cables only. One SINC-positive PHF treated with ORIF sustained a further injury involving a plate fracture, and estimation of subsidence on the follow-up radiograph was not possible. This case was excluded from the statistical analysis.

**Table 1:** Details of PHFs that were SINC positive THR: total hip replacement, CPT: collarless polished tapered, ORIF: open reduction and internal fixation, SINC: subsidence-in-centraliser, PHF: Periprosthetic hip fracture.

Age at the time of injury (years)	Implant	Fixation	Stem	Side	Pre-injury film available	Treatment	Subsidence at latest f/u (mm)	Latest f/u (months)	Reoperation	Notes
61	THR	Hybrid	CPT	R	N	Conservative	4	12	Y	Failed conservative management then revised
86	THR	Hybrid	CPT	R	N	Conservative	4	2		
90	Hemi	Cemented	CPT	L	N	Conservative	3	3		
89	THR	Cemented	CPT	L	Y	ORIF	5	5		
87	THR	Hybrid	CPT	L	Y	ORIF	3	6		
81	THR	Cemented	CPT	R	Y	ORIF	6	10		
81	THR	Hybrid	CPT	L	Y	ORIF	7	16		
81	THR	Cemented	CPT	R	Y	ORIF	9	35		
93	THR	Cemented	CPT	L	Y	ORIF	5	5		
60	THR	Cemented	CPT	L	Y	ORIF	1	2		
80	THR	Cemented	CPT	R	Y	ORIF	5	23		
63	THR	Cemented	CPT	R	Y	ORIF	6	13		
84	THR	Hybrid	C Stem	L	Y	ORIF	6	6	Y	
78	THR	Hybrid	CPT	R	Y	ORIF	5	12		
65	THR	Hybrid	Exeter	L	Y	ORIF	14	23	Y	Plate fracture and failure
75	THR	Cemented	CPT	R	Y	ORIF	5	39		
81	THR	Hybrid	Exeter	R	Y	ORIF	2	3		
50	THR	Cemented	CPT	R	Y	ORIF	5	3		
87	Hemi	Cemented	Exeter	L	Y	ORIF	3	6		
79	Hemi	Cemented	CPT	L	Y	ORIF	N/A	3	Y	Plate fracture + failure - subsidence value not attainable - excluded
91	THR	Hybrid	CPT	L	Y	Revision	N/A	2		
75	THR	Hybrid	CPT	R	Y	Revision	N/A	48		
89	THR	Cemented	CPT	R	Y	Revision	N/A	5		
90	THR	Hybrid	CPT	R	Y	Revision	N/A			
85	THR	Hybrid	CPT	L	Y	Revision	N/A			
65	THR	Hybrid	CPT	L	Y	Revision	N/A		Y	
90	THR	Hybrid	CPT	L	Y	Revision	N/A			
85	THR	Cemented	CPT	R	Y	Revision	N/A			
76	Hemi	Cemented	CPT	L	Y	Revision	N/A			
91	THR	Hybrid	CPT	R	Y	Revision	N/A			
81	THR	Cemented	CPT	L	Y	Revision	N/A			
58	THR	Hybrid	CPT	L	Y	Revision	N/A			
94	Hemi	Cemented	CPT	R	Y	Revision	N/A			
78	THR	Hybrid	CPT	R	Y	Revision	N/A			
71	THR	Hybrid	CPT	R	Y	Revision	N/A			

Nineteen of nineteen (100%) SINC-positive PHFs that were managed conservatively or with ORIF, with appropriate follow-up radiographs available, demonstrated subsequent stem subsidence (mean 5.2 mm, SD 2.8) at a mean of 11.3 months of follow-up (SD 10.8). Seventeen of seventeen (100%) SINC-positive fractures were managed with fixation subsided (mean 5.4, SD 2.8), with 3/17 resulting in further fixation or arthroplasty. Three of three (100%) of SINC-positive patients managed conservatively subsided, including one patient who underwent further revision surgery due to loosening and pain.

SINC negative

**Figure 5:**
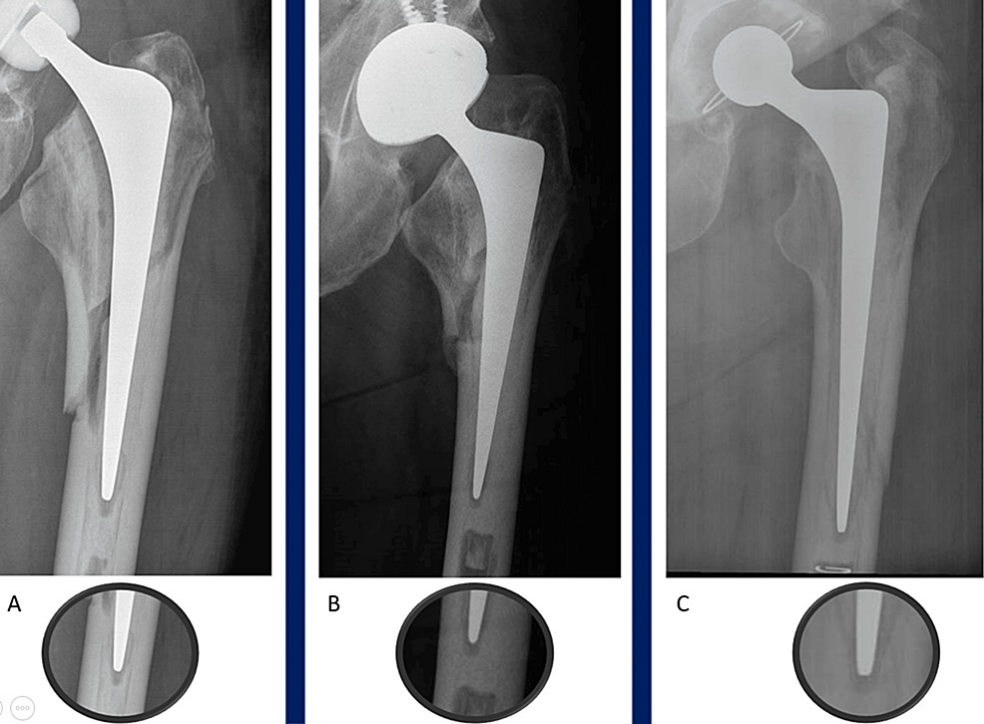
SINC negative periprosthetic femoral fractures AP radiographs of three separate patients (A)-(C) demonstrating periprosthetic hip fractures through the cement mantle with a negative SINC sign. SINC: subsidence-in-centraliser.

Nineteen fractures were deemed to be ‘SINC’ negative (Table 2 and Figure 5). Five of 19 patients were treated conservatively. One patient from this cohort died during their admission. None of the conservatively managed PHFs demonstrated subsequent stem subsidence at a mean follow-up of 12.1 months (SD 9.21). One patient underwent revision arthroplasty with an uncemented distal fix tapered fluted stem.

**Table 2:** Details of PHFs that were SINC negative THR: total hip replacement, CPT: collarless polished tapered, ORIF: open reduction and internal fixation, SINC: subsidence-in-centraliser, PHF: periprosthetic hip fracture.

Age (years)	Implant	Fixation	Stem	Side	Pre-injury film available	Treatment	Subsidence at latest f/u (mm)	Latest f/u (months)	Reoperation	Notes
74	THR	Cemented	CPT	L	Y	Conservative	0	18		
79	THR	Hybrid	CPT	L	Y	Conservative	0	5		
63	THR	Cemented	CPT	L	Y	Conservative	0	24		
77	THR	Hybrid	CPT	R	N	Conservative	0	12		
96	THR	Cemented	CPT	R	Y	Conservative	0	1.5		
57	THR	Cemented	CPT	R	Y	ORIF	1	36		
63	THR	Hybrid	CPT	L	Y	ORIF	0	2		
84	THR	Cemented	C Stem	L	N	ORIF	0	3		
96	THR	Hybrid	CPT	R	N	ORIF	0	9		
71	THR	Hybrid	CPT	L	Y	ORIF	0	6		
58	THR	Hybrid	CPT	L	N	ORIF	0	2		
77	THR	Hybrid	CPT	L	N	ORIF	0	53		
50	THR	Hybrid	C Stem	L	N	ORIF	0	6		
85	THR	Cemented	CPT	L	Y	ORIF	0	6	Y	Plate fatigue failure
61	THR	Cemented	Exeter	L	N	ORIF	0	12		
81	THR	Cemented	CPT	L	N	ORIF	0	24	Y	ORIF with plate and strut graft. Non-union revised with further plating + united. No subsidence
81	THR	Hybrid	CPT	L	N	ORIF	4	26		
59	THR	Cemented	CPT	R	Y	ORIF	0	11		
89	THR	Hybrid	CPT	L	N	Revision arthroplasty	NA	3		

Thirteen of nineteen (68.4%) SINC-negative patients were treated with ORIF. Twelve patients underwent fixation with a single lateral locking plate, including two with supplementary strut grafts, and one patient was treated with cerclage cables alone. Two of thirteen (15%) of these fractures demonstrated subsequent stem subsidence, with a mean subsidence of 0.28 mm (SD 1.1) and a mean follow-up of 15.1 months (SD 9.8).

Sensitivity and specificity of SINC sign

The SINC sign demonstrated a sensitivity of 90.5% in the prediction of subsequent stem subsidence (95% CI 69.6-98.8) and a positive predictive value (PPV) of 100% (Table 3). The SINC sign demonstrated a specificity of 100% (95% CI 79.4-100.0) and a negative predictive value (NPV) of 88.9%. Comparison of the degree of subsequent stem subsidence demonstrated significantly more in the SINC-positive group, U = 6.50 mm, p<0.01 (Table 4).

**Table 3:** 2 × 2 contingency table for sensitivity and specificity of SINC sign FP: false positive; FN: false negative; TP: true positive; TN: true negative, SINC: subsidence-in-centraliser

		Subsequent subsidence		Total
		No	Yes	
SINC	-	16	2	18
		TN	FN	
	+	0	19	19
		TN	TP	
Total		16	21	37
Phi		0.897		
Sensitivity		90.5		
Specificity		100		

**Table 4:** Comparison of mean subsidence between SINC positive and SINC negative groups using the Mann-Whitney U test MWU: Mann-Whitney U test, SINC: subsidence-in-centraliser.

	SINC	N = 37	Mean rank	MWU
Subsidence	Positive	19	27.66	U=6.50
	Negative	18	9.86	P<0.01

Inter- and intra-rater reliability

The SINC sign demonstrated strong inter-rater reliability when measured with two independent assessors, with Kappa 0.96 (95% CI 0.89-1.03). Intra-rater reliability was also found to be strong, with Kappa = 0.86 (95% CI 0.73-0.99). Measurement of subsequent stem subsidence demonstrated strong inter-rater reliability (ICC 0.9).

## Discussion

This study has demonstrated that the SINC sign is associated with a high level of sensitivity (90.5%) and specificity (100%) for the detection of subsequent subsidence in periprosthetic fractures around PTS that are treated without revision surgery. When comparing the degree of subsidence, SINC positive fractures underwent significantly more subsidence compared with SINC negative fractures when fixed (5.4 mm vs. 0.28mm, U = 6.50, p<0.001) at a mean follow-up of 12.7 months.

As such, this tool may serve as a useful adjunct to the existing diagnostic armamentarium for identifying PTS stems that will fare worse with fixation. We propose that SINC-negative PHFs are equivalent to Vancouver B1 fractures and can be safely treated with fixation. By contrast, SINC-positive fractures could be considered for revision arthroplasty either to a distal-fit uncemented stem [25,26] or a cement-in-cement revision [27] in the medically fit patient.

We have previously described the SINC sign, noting that this phenomenon has been alluded to in the literature before [23]. Grammatopoulos et al. noted in a case series of 21 fractures around polished tapered stems that such fractures displayed particular radiographic patterns and that subsidence into the centraliser on radiographs was associated with unstable fractures [28]. However, to the best of our knowledge, this is the first study that quantitatively assesses the association between subsidence into the centraliser and subsequent stem subsidence.

All cemented PTS are associated with some degree of subsidence within the cement mantle [29]. The majority of subsidence in PTS usually occurs within the first two years following surgery, then slows down significantly [30], though Ling et al. [31] and Carrington et al. [32] reported that the Exeter stem can continue to migrate throughout its lifespan [31,32]. This slow, controlled subsidence process is in contrast, however, with rapid subsidence, which can be associated with an increased risk of failure and loosening [30,33,34]. We set out to determine the presence of the SINC sign amongst patients who have undergone THA with PTS without any evidence of fracture. Only 2/107 patient radiographs reviewed demonstrated a positive SINC sign at a mean follow-up of 19.6 months. This was also evident on post-operative films and may have resulted from the impaction of the stem with a mallet during cementation, causing the stem to sink within the centraliser. The absence of the SINC sign in the rest of the cohort suggests that it is not a normal postoperative finding and is not consistent with the natural history of controlled stem subsidence.

It is well documented that the underlying principle of PTS fixation involves controlled subsidence within the cement mantle leading to hoop stress generation at the cement-bone interface and that PTS stems do not achieve true fixation at the cement-stem interface [35]. Some authors have suggested that due to this principle, a fracture through the cement mantle renders the stem 'loose'. In a recent retrospective study of 87 PHFs around PTS, Maggs et al. classified these as either B2W (well fixed) or B2L (loose), depending on the fixation of the cement/bone interface [27]. They conceptualise the PTS prosthesis as including the cement mantle. When the cement fixation to the bone is disrupted, the prosthesis can be thought of as "loose". Scott et al. recently determined that B-type PHFs around PTS stems could be successfully treated with ORIF when the bone cement interface was intact and the fracture was anatomically reducible, postulating in three patients that subsidence of the stem into the centraliser could inhibit this reduction [11]. We theorise that the SINC sign likely indicates a cement mantle that is disrupted such that it cannot be adequately reconstituted or reduced before fixation. Sixteen of eighteen (89.0%) SINC-negative fractures were treated conservatively or with fixation, demonstrating no subsequent subsidence, suggesting that these cement mantles were reducible and therefore fixable with a favourable radiographic outcome. It is clear that the Vancouver classification and treatment algorithm works well for uncemented and composite beam-type stems; however, the lack of clarity surrounding PHFs in PTS highlights the potential benefit of the SINC sign for guiding treatment in this cohort.

The strong inter- and intra-rater reliability demonstrated in this study gives us confidence that the SINC sign is a simple and reproducible radiographic sign that could be utilised by the majority of orthopaedic surgeons with minimal training. Further data on a larger scale using surgeons from multiple centres and of differing experience levels would be useful in further assessing the reproducibility of the sign.

A negative SINC sign demonstrated an 89% negative predictive value for subsequent stem subsidence. It is notable that SINC-positive fractures were more likely to undergo revision surgery compared with SINC-negative (41% vs. 10%), suggesting that the SINC sign correlates with other clinical and radiographic features that led surgeons to opt for revision arthroplasty rather than fixation.

The reoperation rate for PHFs treated with fixation was 23.5% in the SINC-positive group versus 15.4% in the SINC-negative group, and whilst statistical analysis was not performed for this comparison due to a lack of power, this may represent a higher likelihood of failure as a result of symptomatic stem subsidence.

Limitations

Our study is subject to several limitations. A sizeable proportion of patients (6) were lost to follow-up following the management of their PHF. Regular clinics and radiographs are not always possible for this group of frail, elderly patients [5,6]. The SINC sign is only applicable to PTS and not to composite beam stem designs, which account for a significant minority of stems [19]. The use of subsidence (both the rate of and the degree of subsequent stem subsidence) as the main outcome measure has its own limitations. Whilst subsidence is associated with higher reoperation rates and is considered a feature of a failing stem [34,36], there is a lack of evidence evaluating the association between subsidence, pain, and functional outcomes, particularly in modern PTS stems. An asymptomatic subsiding stem does not necessarily equate to a failed construct. Finally, the rule counting stems that had subsided more than 50% as SINC positive was arbitrary but reflected our experience that this constituted a definite visible change in the appearance of the centraliser on radiographs that could not be attributed to rotation or projection.

The sample size used to calculate sensitivity and specificity is not large enough to make conclusive statements. Larger, multi-centre studies with longer clinical, functional, and radiological follow-up would better establish the validity of our findings and the consequent effect on outcomes. This study includes different stem designs, different indications for index arthroplasty, and different fixation methods for those treated with ORIF. Different rates of PHF have been documented for different stem types [18,37], indicating an additional source of bias, controlling for which was precluded in our analysis by the low sample size. The degree of subsidence was measured using non-validated techniques; however, this was applied uniformly to all cases and validated using ICC. It should also be noted that whilst other authors have measured subsidence of the stem in the cement mantle by comparing the final radiograph with the pre-fracture radiograph, we have chosen to measure the subsidence from the injury radiograph to reduce the risk of over-reporting subsidence.

## Conclusions

The SINC sign can serve as a useful adjunct in the decision to fix or revise PHF around PTS. A positive SINC sign may represent a cement mantle that cannot be reconstituted anatomically, leading to subsidence after treatment with ORIF. This sign demonstrates strong inter-rater reliability and is a simple, reproducible tool. The SINC sign may indicate poorer outcomes with osteosynthesis, but further, larger-scale studies with longer-term follow-up, patient-reported outcome data, and additional markers of failure are required to confirm these findings and allow incorporation into treatment algorithms for PHFs.
